# Indicators of young women’s modern contraceptive use in Burkina Faso and Mali from Demographic and Health Survey data

**DOI:** 10.1186/s40834-017-0053-6

**Published:** 2017-11-03

**Authors:** Amy O’Regan, Gretchen Thompson

**Affiliations:** FHI 360, 359 Blackwell St. Suite 200, Durham, NC USA

**Keywords:** Contraceptives, Burkina Faso, Mali, Adolescents

## Abstract

**Background:**

High total fertility rates in Burkina Faso and Mali are leading to population growth beyond the agricultural and fiscal means of its citizens. Providing access to affordable family planning methods is a key step in driving the demographic transition where fertility and mortality rates decline. Furthermore, both nations face significant challenges as climate change is projected to disproportionately impact the western Sahel region undermining environmental, social and economic stability within the region. This analysis was included in formative research to inform family planning programming. The aim of this study was to examine possible indicators of long acting and permanent contraceptive method (LAPM) and short-term method (STM) use for young women in Burkina Faso and Mali.

**Methods:**

Secondary data analysis was conducted using the three most recent Demographic and Health Survey (DHS) datasets for Burkina Faso (1998, 2003, 2010) and Mali (2001, 2006, 2012). Women ages 15–24, at risk for unwanted pregnancy were included in these analyses. Summary descriptive statistics across all time points are reported and multinomial logistic regression was used with the most recent data to determine potential indicators of different types of modern contraceptive methods.

**Results:**

In Burkina Faso in 2010, 24% of women ages 15–24 were using modern contraceptives. Only 2.9% reported using LAPMs in 2010, an increase from 0.3% in 1998. In Mali, modern contraceptive use increased more recently, rising from 9.4% in 2001 to 10.2% in 2006 to 15.3% in 2012. LAPM use also increased from 0.3% in 2001 to 4.1% in 2012. Significant indicators of LAPM contraceptive use in both countries included educational attainment, ideal family size, home ownership and husband’s desire for more children.

**Conclusions:**

Young women in Burkina Faso and Mali are increasingly using modern contraceptives for family planning; however, the LAPM contraceptive prevalence rate remains low. Our analysis indicates that social norms around ideal family size for both men and women continue to drive young women’s choices around family planning and impede use of LAPMs. To increase modern contraceptive use and curb fertility rates, local governments and development organizations should focus on women’s empowerment and include male partners.

**Electronic supplementary material:**

The online version of this article (10.1186/s40834-017-0053-6) contains supplementary material, which is available to authorized users.

## Background

If current projections hold true, the population of Sub-Saharan Africa will more than double in the next 36 years. [1] These significant population increases are particularly apparent in the Sahel region of Africa where the Sahara Desert meets the Savannah plains. Countries in this arid, rural region, have some of the highest total fertility rates (TFR) in the world, ranging from 4.1 in Mauritania to 7.6 in Niger in 2014 [2]. A rapidly increasing population is particularly troubling for this region, where the impact of climate change on agricultural production has already led to substantial food insecurity and is expected to worsen in the future. [1] Maternal, child and neonatal mortality rates in this region rank as some of the highest in the world [2], contributing to a vast number of preventable deaths. Providing access to affordable family planning methods is a key step in driving countries toward a demographic transition where fertility and mortality rates decline. [1]

Although there has been an increase in family planning and contraceptive use over the last few decades, the number of women using modern contraceptives remains low in many countries in the Sahel, contributing to high fertility rates. In Mali, the national TFR was 6.1 in 2014 [2]. In Burkina Faso, it was only slightly lower at 5.9 [2]. High fertility rates can have a devastating impact on women’s health, including increased maternal mortality, birth complications, and unsafe abortions. [3]

Young women in the Sahel likely face significant barriers to accessing family planning methods. Poor health infrastructure, lack of trained providers and contraceptive commodities, financial barriers, and community opposition to family planning can all impede access to modern contraceptives. [4] Prior research shows that wealth can contribute to significant inequities in access and uptake of long-acting and permanent method (LAPM) contraceptives [5], but these studies have not included messaging exposure, sexual and reproductive health indicators, or normative indicators that drive family size and fertility choices. Also, structural and cultural barriers to effective contraceptive use may also be exacerbated for adolescents living in rural areas, and limitations may be particularly acute for those who are unmarried and/or nulliparous.

This paper presents an analysis of Demographic and Health Survey (DHS) data from Burkina Faso and Mali. This analysis was included in formative research to inform family planning programming. The aim of this study was to examine possible indicators of LAPM and short-term method (STM) use for young, sexually active women in Burkina Faso and Mali. We used a range of indicators including personal sexual behavior often missing from determinants of contraceptive use studies [6]. These findings may be used to influence future programming for young women in these countries targeted at increasing LAPM contraceptive uptake.

## Methods

### Data sources

Secondary analysis was conducted using women’s survey data from the DHS for Burkina Faso (1998, 2003, 2010) and Mali (2001, 2006, 2012). Two-stage cluster sampling methods were used to collect data for all countries and time points. Detailed descriptions of sampling methods are described in each of the DHS final reports. [7] The data were weighted to adjust for sampling design and non-response.

### Study design

This study employs a four-tiered approach to examine indicators of LAPM and STM among young women in Burkina Faso and Mali.

1. To measure frequencies and observe trends in contraceptive use and potential indicators, we conducted summary descriptive analysis of women’s survey data from the three most recent DHS for Burkina Faso (1998, 2003, 2010) and Mali (2001, 2006, 2012).
2. Variables were selected for model inclusion based on relevance in the peer-reviewed literature and pared down based on a bivariate correlation analysis and preliminary significance testing (detailed below) to limit the number of co-variates in the model to meet the degrees of freedom threshold required to perform multinomial modeling.
3. Using the most recent data for each country, binomial logistic regression procedures were performed to identify indicators of modern contraceptive use for both married and unmarried women and inform variable inclusion for multinomial models.
4. Using the most recent data for Burkina Faso and Mali, multinomial logistic regression procedures were performed to identify indicators of LAPM and STM for all women and married women.

### Variables

Two primary outcomes were created to describe current contraceptive use. The first binary outcome classifies current contraceptive use as modern methods vs. traditional methods or nonusers. This outcome was used in initial significance testing. For the purposes of these analyses, modern contraceptives include: intra-uterine device (IUD), implant, pill, injection, male sterilization, condom, diaphragm, female condom, foam, or jelly. The second multinomial regression analysis used a three-category outcome variable. Outcome categories were (1) LAPM, which classifies current contraceptive use as long-acting or permanent methods (IUD, implant, male sterilization) vs. (2) short-term modern methods (pill, injection, condom, diaphragm, female condom, foam, or jelly) vs. (3 – reference category) traditional or nonusers.

Variables describing sexual and reproductive health (SRH) behaviors, access to care, contraceptive knowledge, contraceptive use, and fertility intentions were described across all datasets in which indicators were available. Selected variables are listed in Table 1. Frequencies, weighted percentages, means, and 95% confidence intervals were calculated for all variables and are included in an additional file for each country (see Additional files 1 and 2). Response categories for the following variables were collapsed to mitigate the effects of low cell counts during analysis: educational attainment, home ownership, land ownership, religion, marital status, knowledge of contraceptive method, health care decision making, total number of wives, and recent sexual activity. Wealth index, provided by the DHS, was standardized to provide more meaningful interpretations while preserving variability. Standardizing the wealth index was necessary to include wealth as a continuous variable as opposed to one measured ordinally through wealth quintiles.

**Table 1:** Independent variables considered for multivariate model inclusion

**Sociodemographic determinants**	**Access to care**
Wealth index (standardized)	Barrier to seeking medical care: getting permission
Age	
Educational attainment	Barrier to seeking medical care: money
Residence (urban/rural)	Barrier to seeking medical care: distance
Currently working	Barrier to seeking medical care: not wanting to go alone
Religion	**Reproductive Preferences**
Marital status	Ever terminated pregnancy Age at first sex
**Women’s empowerment**	Number of children
Home ownership	Ideal number of children
Land ownership	Desire for more children
*Health care decision making*	*Total number of wives*
Wife beating justified for refusal to have sex	*Age at first marriage/cohabitation* ^a^
**Knowledge and information on family planning methods**	*Age at first birth*
Knowledge of contraceptive method^a^	*Partner’s desire for more children*
Heard family planning messages on radio	**Sexual and Reproductive Health Behavior**
Saw family planning messages in newspaper/magazine	Sex partners, last 12 months
Saw family planning messages on TV	Sex partners, lifetime
Visited by a family planning health care worker in the last 12 months	Recent sexual activity
Visited a health facility in the last 12 months	Contraceptive use and intention^a^
	Last source of modern contraceptive^a^
	Reason for discontinuation of previous modern method^a^

^a^Variables excluded from regression models due to potential multicollinearity, not enough responses or theoretical redundancy with other indicators.

*Italicized variables are specific to ever married women or women with children*

### Data analysis

Descriptive analysis and primary regression analysis was restricted to women ages 15–24 who were at risk of unwanted pregnancy. Women were excluded from the analysis if they reported never having sex, current pregnancy, wanting a child within two years, postpartum amenorrhea, sterilization or infecundity. Women who had missing values for exclusion categories were considered missing for analysis. A second regression analysis was restricted to married or in union women ages 15–24 who were not currently pregnant. The unweighted sample size for each dataset and sub population is detailed in Table 2.

**Table 2:** Unweighted sample size for all datasets and subpopulations

Country	Year	Total sample size	All women included in analysis^a^	Married women included in analysis^a^	Excluded from analysis for missing values
Burkina Faso	2010	17,087	2826	2117	34
	2003	12,477	2130	1505	29
	1998	6445	1139	856	5
Mali	2012	10,424	1485	1093	0
	2006	14,583	2313	2165	88
	2001	12,849	2228	1750	65

^a^Women were excluded from the analysis if they reported never having sex, current pregnancy, wanting a child within two years, postpartum amenorrhea, sterilization or infecundity

All data analysis was conducted using SAS Enterprise Guide Version 7.1. Survey procedures were used to account for complex survey design. Weighting, stratification and clustering variables created by the DHS were used throughout analysis. Standard errors were linearized using the Taylor series linearization method to further account for the complex sample design of the DHS. [8]

To limit redundancy in the multivariate models, variables were pared down based on their correlation coefficient, restricting from the models variables correlated 0.8 or higher and thereby reducing multicollinearity. [9] One variable, age at first marriage, was removed using this method as it was highly correlated with age at first sex and age at first birth. Any variables that were directed to women who use modern contraceptives were not added to the models as they would be too highly correlated with the outcome. This final list of variables used for the modelling was separated into six distinct groups used for the initial analyses (see Table 1).

First, to determine which variables would be included in the models, each group of variables (listed above) was modeled to the binary outcome user via logistic regression to determine significance of associations. Using the type three analysis of effects, which measures how much variables contribute to model fit [10], variables were included in subsequent models if they were significant at *p* ≤ 0.20. All variables that were significant in the initial analyses were included in the final model for the binary outcome user. These analyses were repeated for both countries independently (see Additional file 3). All variables that were significant in the final user model (p ≤ 0.20, type three analysis of effects), were included in the final multinomial logistic regression LAPM model. For all final models, significant variables are reported in Tables 4 and 5 with footnotes listing all insignificant variables included as covariates.

Since some of the variables only applied to married women, the entire multivariate analysis, detailed above, was repeated but restricted to married or in union women. In married women exclusive models, applicable variables (italicized in Table 1) were added to the reproductive preferences and women’s empowerment groups and marital status was removed from the sociodemographic determinants group.

## Results

### Sample description

**Fig. 1:**
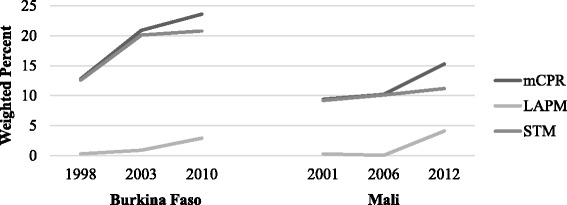
Modern contraceptive use, sexually active women ages 15–24: Trends in the DHS

Modern contraceptive use among young women, although still uncommon, increased in both countries over the time period examined. In Burkina Faso in 2010, the modern contraceptive prevalence rate (mCPR) was 23.6% for women in this sample. Only 2.9% reported using LAPM in Burkina Faso in 2010, an increase from a meager 0.3% in 1998 (Fig. 1). In Mali, the mCPR increased more recently, rising from 9.4% in 2001 to 10.2% in 2006 to 15.3% in 2012. LAPM use also increased from 0.3% in 2001 to 4.1% in 2012 (Fig. 1).

Knowledge of a modern family planning method is high in both countries, ranging from 89.5% in Mali to 97.8% in Burkina Faso in the most recent DHS data. Age at first sex, first marriage, first birth, and ideal number of children were consistent over the course of the examined time-period. Most young women in this region married and reached sexual debut between the ages of 15 and 16. In both Burkina Faso and Mali, women considered the ideal number of children to be between 5 and 6. Less than 7% of women in these populations reported ever having terminated a pregnancy.^1^Unmet need for family planning was high among this subset of the population in Mali at 49.8% in 2012, and it had increased from 42.2% in 2001. Burkina Faso saw a modest decline in unmet need for this subpopulation from 33.8% in 1998 to 30.9% in 2010 (Table 3).

**Table 3:** Overview of key SRH indicators (weighted percentages and means)

	Burkina Faso			Mali		
	1998	2003	2010	2001	2006	2012
mCPR	12.8	20.9	23.6	9.4	10.2	15.3
LAPM use	0.3	0.9	2.9	0.3	0.04	4.1
STM use	12.6	20.1	20.8	9.2	10.1	11.2
Knowledge of modern FP	79.6	92.2	97.8	81.4	81.2	89.5
Age at first sex (mean)	16.1	16.4	16.5	15.2	15.4	15.5
Age at marriage (mean)	16.5	16.8	16.6	15.5	15.5	15.6
Age at first birth (mean)	17.8	18.0	18.0	17.0	17.0	16.8
Ideal no. of children (mean)	5.3	5.0	4.9	5.4	5.4	5.3
Unmet need for FP	33.8	32.2	30.9	42.2	45.9	49.8
Ever terminated pregnancy	#	5.4	5.3	6.6	6.8	3.9

# Question not asked in survey

### Multinomial analysis

In Burkina Faso, age, home ownership, having two lifetime sex partners, reporting fewer ideal number of children and being sexually active in the last four weeks were significantly associated with the use of LAPM in both models (*p* ≤ 0.05). Being from a rural area, reduced the odds of LAPM by 80% compared to being from an urban area (*p* ≤ 0.05). For each additional child the woman reported as the ideal family size, the odds of using LAPM decreased by 20% for all women and 30% for married women. Women who reported owning a home, either alone or jointly, had twice the odds of using a LAPM (*p* ≤ 0.05).

Much like LAPM, age, home ownership, lower ideal number of children, having two lifetime sex partners, and being sexually active in the last four weeks were associated with use of STM in both models (p ≤ 0.05). In addition, secondary educational attainment was positively associated with use of STM for both models (p ≤ 0.05). Completing primary school increased the odds of STM use by 40% in the inclusive model (*p* ≤ 0.05), but did not remain significant when limited to married women. In both models, the odds of using STM was 30% less for women who reported that money was a big problem related to getting medical help. The odds of using STM was 2.1 times greater for all nulliparous women and 3 times greater for nulliparous married women, compared to those with children.

Women who were not in union or living with a partner outside of marriage had 1.8 times higher odds of using STMs (*p* ≤ 0.05) compared to married women. In the inclusive model (all women), seeing family planning messaging on TV and having three or more lifetime sex partners was associated with STM use (*p* ≤ 0.05). Women had 50% lower odds of using STMs if they reported that their husband wanted more children than they did and 40% lower odds if they did not know how many children their husbands wanted (*p* ≤ 0.05) (Table 4).

**Table 4:** Burkina Faso 2010: Variables showing evidence of association with LAPM and STM

	All women *N* = 2826 OR (95% CI)^†^			Married women *N* = 2177 OR(95% CI)^†^	
	Percent or mean	LAPM	STM	LAPM	STM
Wealth Index (standardized)	*N/A*	*N/A*		1.1 (0.8, 1.5)	1.5 (1.1, 1.9)*
Age	20.7 (mean)	1.1 (1.0, 1.3)*	1.1 (1.0, 1.1)*	1.1 (1, 1.3)	1.1 (1, 1.2)*
Educational attainment					
No education	64.2	REF	REF	REF	REF
Primary	17.0	1.2 (0.6, 2.4)	1.5 (1.1, 2.1)*	1 (0.5, 2.1)	1.2 (0.8, 1.8)
Secondary or higher	18.8	2.3 (1, 5.6)	3 (2.1, 4.2)*	2.1 (0.9, 5.3)	2.3 (1.3, 4)*
Urban/Rural					
Urban	32.5	REF	REF	REF	REF
Rural	67.5	0.2 (0.1, 0.5)*	0.6 (0.4, 0.8)*	0.2 (0.1, 0.5)*	0.7 (0.4, 1.2)
Marital status				*N/A*	
Married	22.5	REF	REF		
Never in union	68.2	0.5 (0.1, 2)	1.8 (1.1, 3.1)*		
Living with partner	6.7	2.3 (1, 5.2)	1.8 (1.1, 2.8)*		
Widowed/Divorced/Separated	2.7	1.9 (0.4, 9.5)	1 (0.3, 3.2)		
Home ownership					
Does not own	75.2	REF	REF	REF	REF
Owns alone or jointly	24.8	2 (1.2, 3.5)*	1.9 (1.4, 2.7)*	1.9 (1.1, 3.3)*	1.9 (1.3, 2.7)*
Saw FP messages on TV				*N/A*	
No	72.7	REF	REF		
Yes	27.3	0.9 (0.5, 1.7)	1.6 (1.1, 2.2)*		
Barrier to getting medical help: money					
Big problem	31.2	1.2 (0.7, 1.9)	0.7 (0.6, 0.9)*	1.1 (0.7, 1.9)	0.7 (0.5, 1.0)*
Small problem	68.8	REF	REF	REF	REF
Respondent has children					
No	23.40	0.4 (0.1, 1.5)	2.1 (1.3, 3.4)*	0.7 (0.1, 3.5)	3.0 (1.4, 3.5)*
Yes	76.60	REF	REF	REF	REF
Ideal number of children	4.9 (mean)	0.8 (0.6, 1.0)*	0.9 (0.8, 1.0)*	0.7 (0.6, 0.9)*	0.9 (0.8, 1.0)*
Husband’s desire for more children		*N/A*			
Both wants same	43.7			REF	REF
Husband wants more	35.9			0.6 (0.3, 1.1)	0.5 (0.3, 0.8)*
Husband wants fewer	3.6			1.9 (0.6, 6.1)	1.3 (0.5, 3.3)
Don’t know	16.8			0.6 (0.2, 1.4)	0.6 (0.3, 1.0)*
Sex partners, lifetime					
1 partner	77.8	REF	REF	REF	REF
2 partners	16.4	2.4 (1.4, 4.2)*	1.4 (1.0, 2.0)*	2.5 (1.3, 4.7)*	1.5 (1.0, 2.4)
3+ partners	5.7	1.2 (0.4, 3.9)	2.2 (1.3, 3.7)*	1.2 (0.3, 5.1)	2.3 (1.2, 4.3)*
Recent sexual activity					
Active in the last 4 weeks	46.3	8.5 (4.4, 16.2)*	5.9 (4.4, 7.9)*	9.3 (4.3, 20.2)*	9.5 (6.3, 14.3)*
Not active in last 4 weeks	53.7	REF	REF	REF	REF

^†^Adjusted for the following variables: currently working, religion, respondent’s desire for children. N/A variable not included in model. *p < 0.05

In Mali, completing secondary school was significantly associated with both LAPM and STM use (*p* ≤ 0.05). In contrast to the findings in Burkina Faso, women who reported home ownership had lower odds of using STM in both models (p ≤ 0.05). Visiting a health facility in the last 12 months was significantly associated with STM use in both models (p ≤ 0.05). In all models, the odds of using STM or LAPM decreased by 20–30% as ideal number of children increased. Married women had 80% lower odds of using an LAPM and 70% lower odds of using a STM if they reported that their husband wanted more children than they did (*p* ≤ 0.05) (Table 5).

**Table 5:** Mali 2012: Variables showing evidence of association with LAPM and STM

	All women *N* = 1485 OR (95% CI)^†^			Married women *N* = 1093 OR (95% CI)^†^	
	Percent or mean	LAPM	STM	LAPM	STM
Age	20.0 (mean)	1.1 (1.0, 1.3)	1.1 (1.0, 1.2)	1.3 (1.1, 1.5)*	1.1 (1, 1.3)
Educational attainment					
No education	59.6	REF	REF	REF	REF
Primary	11.0	1.7 (0.7, 4.1)	0.7 (0.3, 1.5)	1.1 (0.2, 5.2)	1.2 (0.5, 2.7)
Secondary	29.4	2.5 (1.2, 5.4)*	2.6 (1.4, 4.6)*	3.4 (1.3, 8.9)*	4.2 (2.2, 8.1)*
Home ownership					
Does not own	61.5	REF	REF	REF	REF
Owns alone or jointly	38.5	1.0 (0.5, 1.9)	0.5 (0.3, 0.9)*	0.7 (0.4, 1.6)	0.5 (0.3, 0.8)*
Visited health facility in last 12 m					
No	71.9	REF	REF	REF	REF
Yes	28.1	2 (0.9, 4.3)	1.9 (1.2, 2.8)*	1.7 (0.7, 4.0)	1.6 (1, 2.7)*
Respondent’s desire for more children					
Wants after 2+ years	52.7	3.6 (0.8, 17.2)	2.3 (0.9, 5.9)	–	4.2 (1.2, 14.6)*
Wants, unsure timing	39.3	5.5 (1.1, 27.6)*	1.7 (0.6, 4.6)	–	1.2 (0.3, 5.2)
Wants no more or can’t have more	8.1	REF	REF	REF	REF
Ideal number of children	5.3 (mean)	0.8 (0.7, 1)*	0.8 (0.7, 0.9)*	0.7 (0.6, 0.9)*	0.8 (0.7, 0.9)*
Husband’s desire for more children		*N/A*			
Both wants same	17.6			REF	REF
Husband wants more	35.3			0.2 (0.1, 0.5)*	0.3 (0.2, 0.8)*
Husband wants fewer	3.1			0.4 (0.1, 1.8)	0.5 (0.1, 3)
Don’t know	44.0			0.2 (0.1, 0.6)*	0.5 (0.3, 1.1)
Lifetime sex partners				*N/A*	
1 partner	80.7	REF	REF		
2 partners	12.9	1.5 (0.8, 2.7)	1.9 (1.1, 3.4)*		
3+ partners	6.5	0.8 (0.3, 1.9)	2.4 (1.2, 4.9)*		
Recent sexual activity					
Active in the last 4 weeks	61.6	2.3 (1.3, 3.9)*	1.8 (1.2, 2.9)*	2.2 (0.9, 5.7)	1.2 (0.7, 2.2)
Not active in last 4 weeks	38.4	REF	REF	REF	REF

^†^Adjusted for the following variables: religion, getting medical help: not wanting to go alone, number of other wives. N/A variable not included in model. *p < 0.05

## Discussion

The descriptive analysis of DHS data offers insights into promising trends for the demand and uptake of LAPM and STM contraceptive use among young women throughout Burkina Faso and Mali. Modern contraceptive use, including LAPM, are increasing in both countries, along with knowledge of modern contraceptives. The United Nations predicts that the average mCPR will increase from 17% in 2015 to 24% in 2030 in West Africa. [11] Although these trends mean more women will use modern contraceptives, they are still too low to meet the Sustainable Development Goals [12] and it may not have a discernable impact on the TFR.

Our analysis shows that young women are still marrying at early adolescence with very little change over time. Women seem to have their first child within a year of first marriage. The literature suggests this is often because young women must prove their fertility before using any type of hormonal contraceptive [13]. However, our analyses show that nulliparous women had increased odds of using STM in Burkina Faso, however there was no effect on LAPM use. This may indicate that women perceive increased risk of infertility from LAPMs and perceive STMs to be safer. Myths about the safety of hormonal contraceptives for women, and long-acting methods in particular, must be dispelled to provide real family planning options to nulliparous women.

Women in both countries reported high ideal numbers of children, which was a statistically significant indicator in all multinomial models in both countries. We also found that partner’s desired family size was significantly associated with contraceptive use. Married young women who reported that their husbands wanted more children than they did had lower odds of using STM in Burkina Faso and both STM and LAPM in Mali. This is consistent with the literature that finds a husband’s reproductive preferences is a determinant of reproductive behavior. [14, 15] In both Burkina Faso and Mali, the ideal number of children is high, with women reporting they wanted to have five children on average, and the husband’s desire for more children is significantly associated with contraceptive use. Thus, it is reasonable young women would often not be interested in using LAPM, even though they are aware of it.

Similarly, young women who reported home ownership (either alone or jointly) in comparison to not owning a home had higher odds of using STM and LAPM in Burkina Faso. This may be a proxy for women’s empowerment and wealth in the sense that if a woman has real or perceived ownership of her home, she may have more power in the home and over her reproductive choices. Changing norms to give women more ownership of physical property may translate into empowerment over their own family planning.

Our results indicate that several key indicators may not play a significant role in modern contraceptive use. Access to care variables and knowledge indicators were mostly not associated with contraceptive use. Although some women may be too young to have sought care, these findings reinforce the notion that young women may not use contraceptives because of social norms and familial circumstances that prescribe their reproductive choices. In order to empower women to make their own reproductive choices, we must work to change societal norms that may limit their personal agency. [16]

Since these results are cross-sectional, we are limited in our ability to infer causation and can only attest to associations between contraceptive use and potential indicators. Also, given the rarity of our outcome, the number of covariates we could include in the models was limited to what would allow for convergence. Therefore, we may not have controlled for all possible confounding factors. Regardless of these limitations, our findings are generally consistent with the peer-reviewed literature.

## Conclusion

Young women in Burkina Faso and Mali are increasingly using LAPM and STM contraceptives for family planning. However, these increases are not at the rate needed to meet the United Nations’ Sustainable Development Goals or to have an impact on the resource-depleting population boom both countries are experiencing. In a society where ideal family size is high and male spousal reproductive preferences are honored, social norms may limit women’s choices around family planning and use of modern contraceptives. To increase modern contraceptive use and curb fertility rates, local governments and development organizations should focus on women’s empowerment and include male partners, as their preferences matter when women make reproductive choices.

## Additional files

Additional file 1: Burkina Faso: Distribution of variables of interest for sexually active women ages 15–24: Trends in the DHS Women’s Survey. This document includes a table of frequencies and weighted percentages for all variables of interest in Burkina Faso for the three most recent DHS surveys. (DOCX 35 kb)

Additional file 2: Mali: Distribution of variables of interest for sexually active women ages 15–24: Trends in the DHS Women’s Survey. This document includes a table of frequencies and weighted percentages for all variables of interest in Mali for the three most recent DHS surveys. (DOCX 34 kb)

Additional file 3: Binary outcome analysis. This document includes a description of preliminary significance testing using a binary outcome to limit variables for model inclusion in the multivariate analysis. (DOCX 18 kb)

## Abbreviations

DHS: Demographic and health survey
IUD: Intra-uterine device
LAPM: Long acting or permanent method
mCPR: Modern contraceptive prevalence rate
SRH: Sexual and Reproductive Health
STM: Short term method
TFR: Total fertility rate

## Footnotes

1 These results may be impacted by the Mexico city policy and other international and local abortion laws. This was not the focus of this study and the impact of abortion policy should be explored in future research.
