# Supplementary material for: Indicators of young women’s modern contraceptive use in Burkina Faso and Mali from Demographic and Health Survey data
Source: Contracept Reprod Med. 2017 Nov 3;2:26. doi: 10.1186/s40834-017-0053-6 (PMC5683538; doi:10.1186/s40834-017-0053-6)
Supplement: Supplementary file 1 — Burkina Faso: Distribution of variables of interest for sexually active women ages 15–24: Trends in the DHS Women’s Survey. This document includes a table of frequencies and weighted percentages for all variables of interest in Burkina Faso for the three most recent DHS surveys. (DOCX 35 kb) [file 40834_2017_53_MOESM1_ESM.docx]

Burkina Faso: Distribution of variables of interest for sexually active women ages 15-24: Trends in the DHS Women’s Survey

| **Variable Description** | **1998** | | **2003** | | **2010** | |
| --- | --- | --- | --- | --- | --- | --- |
|  | **Unweighted N = 1139** | | **Unweighted N = 2130** | | **Unweighted N = 2826** | |
|  | **Unweighted Frequency** | **Weighted Percent (95% CI)** | **Unweighted Frequency** | **Weighted Percent (95% CI)** | **Unweighted Frequency** | **Weighted Percent (95% CI)** |
| Wealth Index |  |  |  |  |  |  |
| Poorest | *N/A* | | 322 | 16.8 (14.4, 19.2) | 354 | 13.8 (12, 15.6) |
| Poorer |  |  | 364 | 18.3 (15.7, 20.9) | 485 | 18 (16.3, 19.7) |
| Middle |  |  | 449 | 21.8 (19, 24.7) | 510 | 17.9 (16.1, 19.7) |
| Richer |  |  | 338 | 15.5 (12.9, 18.2) | 574 | 19.8 (17.6, 21.9) |
| Richest |  |  | 657 | 27.6 (22.8, 32.3) | 903 | 30.6 (27.7, 33.4) |
| Age (mean and 95% CI) | 20.2 (20.1, 20.4) | | 20.3 (20.2, 20.5) | | 20.7 (20.6, 20.8) | |
| Educational attainment |  |  |  |  |  |  |
| No education | 839 | 79.7 (76.6, 82.9) | 1476 | 71.7 (67.9, 75.6) | 1757 | 64.2 (61.7, 66.6) |
| Primary | 145 | 10.7 (8.6, 12.9) | 342 | 14.7 (12.6, 16.7) | 498 | 17 (15.3, 18.8) |
| Secondary or higher | 155 | 9.5 (7.6, 11.4) | 312 | 13.6 (10.8, 16.3) | 571 | 18.8 (16.7, 20.9) |
| Urban/rural |  |  |  |  |  |  |
| Urban | 355 | 20.8 (18.1, 23.5) | 650 | 26.3 (21, 31.5) | 1064 | 32.5 (29.6, 35.3) |
| Rural | 784 | 79.2 (76.5, 81.9) | 1480 | 73.7 (68.5, 79) | 1762 | 67.5 (64.7, 70.4) |
| Currently working |  |  |  |  |  |  |
| No | 482 | 40.8 (36.3, 45.2) | 427 | 19.3 (16.1, 22.6) | 840 | 30.9 (28.2, 33.6) |
| Yes | 656 | 59.2 (54.8, 63.7) | 1702 | 80.7 (77.4, 83.9) | 1981 | 69.1 (66.4, 71.8) |
| Religion |  |  |  |  |  |  |
| Muslim | 699 | 61.5 (55.3, 67.8) | 1276 | 64.3 (60.6, 68) | 1782 | 64.2 (61.2, 67.1) |
| Christian | 316 | 26 (21.3, 30.7) | 619 | 26.5 (23.3, 29.8) | 862 | 30.1 (27.4, 32.8) |
| Animist/Traditional | 94 | 9.6 (6.4, 12.8) | 194 | 7.7 (5.9, 9.4) | 155 | 4.9 (3.4, 6.4) |
| No religion | 30 | 2.9 (1.5, 4.3) | 40 | 1.5 (0.9, 2.1) | 21 | 0.8 (0.4, 1.3) |
| Marital status |  |  |  |  |  |  |
| Never in union | 283 | 21.3 (18, 24.5) | 625 | 28.1 (24.4, 31.8) | 709 | 22.5 (20.1, 24.9) |
| Married | 752 | 70.7 (66.8, 74.6) | 1218 | 59.2 (55.2, 63.3) | 1860 | 68.2 (65.4, 71) |
| Living with partner | 73 | 5.5 (4, 7) | 228 | 10.3 (8.4, 12.2) | 178 | 6.7 (5.3, 8.1) |
| Widowed, Divorced or Separated | 31 | 2.5 (1.5, 3.4) | 59 | 2.4 (1.6, 3.1) | 79 | 2.7 (2, 3.4) |
| Health decision making |  |  |  |  |  |  |
| Husband alone or someone else | *N/A* | | 1837 | 85.4 (83, 87.7) | 1600 | 78.5 (75.8, 81.1) |
| Respondent alone or with husband |  |  | 293 | 14.6 (12.3, 17) | 436 | 21.5 (18.9, 24.2) |
| Wife beating justified for refusal to have sex |  |  |  |  |  |  |
| No | *N/A* | | 1276 | 62.7 (59.6, 65.7) | 2317 | 81.1 (79.1, 83.1) |
| Yes |  |  | 763 | 37.3 (34.3, 40.4) | 496 | 18.9 (16.9, 20.9) |
| Home ownership |  |  |  |  |  |  |
| Does not own | *N/A* | | *N/A* | | 2132 | 75.2 (72.8, 77.6) |
| Owns alone or jointly |  |  |  |  | 693 | 24.8 (22.4, 27.2) |
| Land ownership |  |  |  |  |  |  |
| Does not own | *N/A* | | *N/A* | | 2130 | 73.9 (71.4, 76.4) |
| Owns alone or jointly |  |  |  |  | 696 | 26.1 (23.6, 28.6) |
| Knowledge of contraceptive method |  |  |  |  |  |  |
| Knows no method | 193 | 19.3 (15.6, 23) | 130 | 6.6 (5.2, 8) | 51 | 2.2 (1.4, 3) |
| Knows only folkloric/traditional method | 11 | 1.2 (0.4, 1.9) | 24 | 1.1 (0.5, 1.7) | 1 | 0 (0, 0.1) |
| Knows modern method | 935 | 79.6 (75.7, 83.4) | 1976 | 92.3 (90.8, 93.7) | 2774 | 97.8 (96.9, 98.6) |
| Heard FP on radio |  |  |  |  |  |  |
| No | 693 | 65.4 (61.3, 69.5) | 1006 | 46.9 (43.9, 49.9) | 1174 | 43.6 (40.8, 46.3) |
| Yes | 446 | 34.6 (30.5, 38.7) | 1124 | 53.1 (50.1, 56.1) | 1652 | 56.4 (53.7, 59.2) |
| Heard FP on TV |  |  |  |  |  |  |
| No | 928 | 86.8 (84.6, 89) | 1598 | 75.4 (71.8, 78.9) | 2000 | 72.7 (70.2, 75.2) |
| Yes | 210 | 13.2 (11, 15.4) | 532 | 24.6 (21.1, 28.2) | 825 | 27.3 (24.8, 29.8) |
| Heard FP in newspaper/magazine |  |  |  |  |  |  |
| No | 1059 | 95.1 (93.6, 96.5) | 1903 | 90 (87.9, 92.1) | 2568 | 91.1 (89.5, 92.6) |
| Yes | 80 | 4.9 (3.5, 6.4) | 227 | 10 (7.9, 12.1) | 257 | 8.9 (7.4, 10.5) |
| Visited by FP worker in last 12m |  |  |  |  |  |  |
| No | 1086 | 95.7 (94.5, 96.9) | 1922 | 89.8 (87.8, 91.8) | 2630 | 92.2 (90.8, 93.7) |
| Yes | 53 | 4.3 (3.1, 5.5) | 206 | 10.2 (8.2, 12.2) | 196 | 7.8 (6.3, 9.2) |
| Visited health facility in last 12m |  |  |  |  |  |  |
| No | 688 | 63.2 (59.5, 66.9) | 1288 | 61.7 (58.7, 64.7) | 822 | 28.9 (26.5, 31.3) |
| Yes | 451 | 36.8 (33.1, 40.5) | 842 | 38.3 (35.3, 41.3) | 2004 | 71.1 (68.7, 73.5) |
| Barrier to getting medical help: permission to go |  |  |  |  |  |  |
| Big problem | *N/A* | | 326 | 15.6 (13.5, 17.8) | 571 | 18.9 (16.6, 21.2) |
| Small problem |  |  | 1803 | 84.4 (82.2, 86.5) | 2254 | 81.1 (78.8, 83.4) |
| Barrier to getting medical help: money |  |  |  |  |  |  |
| Big problem | *N/A* | | 1196 | 57 (54.3, 59.7) | 1958 | 68.8 (66.2, 71.4) |
| Small problem |  |  | 934 | 43 (40.3, 45.7) | 867 | 31.2 (28.6, 33.8) |
| Barrier to getting medical help: distance |  |  |  |  |  |  |
| Big problem | *N/A* | | 868 | 43.3 (39.7, 46.8) | 1107 | 39.8 (36.9, 42.7) |
| Small problem |  |  | 1262 | 56.7 (53.2, 60.3) | 1718 | 60.2 (57.3, 63.1) |
| Barrier to getting medical help: not wanting to go alone |  |  |  |  |  |  |
| Big problem | *N/A* | | 526 | 28.3 (25.5, 31.2) | 490 | 17.1 (15.1, 19) |
| Small problem |  |  | 1604 | 71.7 (68.8, 74.5) | 2333 | 82.9 (81, 84.9) |
| Number of other wives |  |  |  |  |  |  |
| No other wives | 504 | 58.8 (54.8, 62.9) | 968 | 67.3 (63.4, 71.2) | 1483 | 72.2 (69.6, 74.8) |
| 1 other wife | 226 | 28.6 (25.1, 32.1) | 320 | 21.8 (18.9, 24.7) | 429 | 21.9 (19.7, 24.2) |
| 2-3 other wives | 87 | 11.6 (8.9, 14.4) | 146 | 10.3 (8.2, 12.3) | 115 | 5.6 (4.3, 6.8) |
| 4+ other wives | 7 | 0.9 (0.1, 1.7) | 12 | 0.6 (0.2, 1.1) | 8 | 0.3 (0.1, 0.5) |
| Age at first marriage/cohabitation (mean and 95% CI) | 16.5 (16.3, 16.7) | | 16.8 (16.7, 17) | | 16.6 (16.5, 16.7) | |
| Age at first sex (mean and 95% CI) | 16.1 (16, 16.3) | | 16.4 (16.3, 16.5) | | 16.5 (16.4, 16.6) | |
| Respondent has children |  |  |  |  |  |  |
| No | 306 | 24.4 (21.1, 27.7) | 591 | 27.4 (24.1, 30.7) | 688 | 23.4 (21, 25.9) |
| Yes | 833 | 75.6 (72.3, 78.9) | 1539 | 72.6 (69.3, 75.9) | 2138 | 76.6 (74.1, 79) |
| Age at first birth (mean and 95% CI) | 17.8 (17.6, 18) | | 18 (17.9, 18.2) | | 18 (17.9, 18.1) | |
| Number of children (mean and 95% CI) | 1.6 (1.5, 1.6) | | 1.5 (1.5, 1.6) | | 1.6 (1.6, 1.7) | |
| Ideal number of children (mean and 95% CI) | 5.2 (5, 5.5) | | 5.0 (4.9, 5.2) | | 4.9 (4.8, 4.9) | |
| Respondent's desire for more children |  |  |  |  |  |  |
| Wants after 2+ years | 829 | 73.8 (70.8, 76.8) | 1599 | 76.5 (73.4, 79.5) | 2198 | 78.8 (76.6, 81.1) |
| Wants, unsure timing | 238 | 19.7 (16.8, 22.5) | 484 | 21.4 (18.4, 24.4) | 581 | 19.4 (17.2, 21.7) |
| Wants no more or can't have more | 72 | 6.5 (4.8, 8.2) | 47 | 2.1 (1.3, 3) | 47 | 1.8 (1.2, 2.3) |
| Husband's desire for more children |  |  |  |  |  |  |
| Both wants same | 302 | 35.2 (30.9, 39.4) | 467 | 32.9 (29.6, 36.2) | 911 | 43.7 (41, 46.5) |
| Husband wants more | 141 | 17.9 (14.9, 20.9) | 291 | 21.6 (18.6, 24.7) | 743 | 35.9 (33.2, 38.6) |
| Husband wants fewer | 11 | 1.3 (0.5, 2) | 32 | 1.8 (1.1, 2.5) | 69 | 3.6 (2.5, 4.6) |
| Don't know | 370 | 45.7 (40.3, 51) | 652 | 43.7 (40.4, 47) | 313 | 16.8 (14.4, 19.2) |
| Ever terminated pregnancy |  |  |  |  |  |  |
| No | *N/A* | | 2017 | 94.6 (93.2, 96) | 2681 | 94.7 (93.8, 95.6) |
| Yes |  |  | 113 | 5.4 (4, 6.8) | 145 | 5.3 (4.4, 6.2) |
| Sex partners, last 12m |  |  |  |  |  |  |
| 0-1 partner | *N/A* | | 2083 | 97.4 (96.5, 98.4) | 2780 | 98.4 (97.9, 98.9) |
| 2+ Partners |  |  | 45 | 2.6 (1.6, 3.5) | 45 | 1.6 (1.1, 2.1) |
| Sex partners, lifetime |  |  |  |  |  |  |
| 1 partner | *N/A* | | *N/A* | | 2174 | 77.8 (76, 79.7) |
| 2 partners |  |  |  |  | 481 | 16.4 (14.8, 18.1) |
| 3+ partners |  |  |  |  | 168 | 5.7 (4.8, 6.7) |
| Recent sexual activity |  |  |  |  |  |  |
| Active in the last 4 weeks | *N/A* | | 762 | 36.9 (34.2, 39.6) | 1300 | 46.3 (44.2, 48.4) |
| Not active in last 4 weeks |  |  | 1366 | 63.1 (60.4, 65.8) | 1522 | 53.7 (51.6, 55.8) |
| Contraceptive use and intention |  |  |  |  |  |  |
| Using modern method | 186 | 12.8 (10.6, 15.1) | 475 | 21 (17.7, 24.2) | 701 | 23.6 (21.5, 25.8) |
| Using traditional method | 102 | 9.1 (6.9, 11.4) | 126 | 5.7 (4.3, 7) | 29 | 1.1 (0.6, 1.6) |
| Non user intends to use | 491 | 43.6 (39.7, 47.4) | 1064 | 49.6 (46.6, 52.7) | 1625 | 57.2 (54.7, 59.8) |
| Does not intend to use | 360 | 34.5 (30.7, 38.3) | 465 | 23.7 (21.2, 26.3) | 471 | 18 (15.9, 20.1) |
| Last source of modern contraceptive |  |  |  |  |  |  |
| Government clinic/pharmacy | 48 | 25.7 (18.6, 32.8) | 144 | 26.8 (21, 32.5) | 365 | 52.8 (47.2, 58.4) |
| Private clinic/delivery | 7 | 3.5 (0.7, 6.3) | 7 | 0.6 (0, 1.3) | 5 | 0.9 (0, 2) |
| Pharmacy | 22 | 11 (6.1, 15.9) | 82 | 17.5 (11.5, 23.6) | 109 | 17.7 (12.9, 22.5) |
| Shop, church, friend | 98 | 54 (45.5, 62.4) | 221 | 50.6 (43, 58.1) | 213 | 27.7 (23.7, 31.8) |
| Other | 11 | 5.9 (2.1, 9.7) | 16 | 4.5 (2.2, 6.8) | 4 | 0.8 (0, 1.7) |
| Reasons for discontinuation of last method |  |  |  |  |  |  |
| Became pregnant | *N/A* | | *N/A* | | 15 | 6.1 (2.6, 9.6) |
| Wanted to become pregnant |  |  |  |  | 77 | 39 (29.8, 48.2) |
| Husband disapproved |  |  |  |  | 8 | 3.2 (0.6, 5.9) |
| Side effects |  |  |  |  | 39 | 20.1 (13.6, 26.6) |
| Access, availability |  |  |  |  | 4 | 2.8 (0, 6.4) |
| Wanted more effective method |  |  |  |  | 12 | 5.7 (2.4, 9.1) |
| Inconvenient to use |  |  |  |  | 5 | 2.5 (0.1, 4.9) |
| Infrequent sex, husband away |  |  |  |  | 19 | 8.7 (3.1, 14.4) |
| Cost |  |  |  |  | 4 | 2.2 (0, 4.3) |
| Marital dissolution |  |  |  |  | 5 | 3.8 (0.4, 7.2) |
| Other |  |  |  |  | 10 | 5.8 (2, 9.7) |
| Condom use at last sex |  |  |  |  |  |  |
| No | 951 | 87.5 (85.1, 89.9) | 1130 | 74.2 (70.3, 78.1) | 1729 | 77.6 (74.9, 80.3) |
| Yes | 181 | 12.5 (10.1, 14.9) | 401 | 25.8 (21.9, 29.7) | 524 | 22.4 (19.7, 25.1) |
| Current contraceptive method |  |  |  |  |  |  |
| Not using | 851 | 78 (74.7, 81.4) | 1529 | 73.4 (70, 76.7) | 2096 | 75.2 (73, 77.5) |
| Pill | 37 | 2.4 (1.5, 3.4) | 78 | 2.8 (2, 3.6) | 115 | 3.8 (3, 4.5) |
| IUD | 4 | 0.2 (0, 0.4) | 2 | 0.1 (0, 0.2) | 3 | 0.2 (0, 0.3) |
| Injections | 15 | 1.1 (0.5, 1.7) | 43 | 1.8 (1.1, 2.4) | 167 | 6.1 (4.9, 7.2) |
| Diaphragm | 3 | 0.2 (0, 0.4) | 0 | 0.0 (0.0, 0.0) | 0 | 0.0 (0.0, 0.0) |
| Condom | 126 | 8.9 (7, 10.7) | 326 | 15.5 (12.6, 18.3) | 321 | 10.7 (9, 12.4) |
| Male sterilization | 0 | 0.0 (0.0, 0.0) | 0 | 0.0 (0.0, 0.0) | 0 | 0.0 (0.0, 0.0) |
| Periodic abstinence | 78 | 6.8 (4.8, 8.8) | 77 | 3.4 (2.5, 4.3) | 24 | 0.9 (0.5, 1.3) |
| Withdrawl | 5 | 0.4 (0, 0.7) | 3 | 0.1 (0, 0.4) | 3 | 0.1 (0, 0.3) |
| Other traditional | 1 | 0.1 (0, 0.3) | 2 | 0.2 (0, 0.5) | 2 | 0.1 (0, 0.3) |
| Implants/norplant | 1 | 0.1 (0, 0.1) | 25 | 0.9 (0.4, 1.3) | 91 | 2.7 (2, 3.4) |
| Abstinence | 18 | 1.9 (0.6, 3.1) | 44 | 1.9 (0.9, 2.9) | 0 | 0.0 (0.0, 0.0) |
| Female condom | 0 | 0.0 (0.0, 0.0) | 0 | 0.0 (0.0, 0.0) | 0 | 0.0 (0.0, 0.0) |
| Foam or jelly | 0 | 0.0 (0.0, 0.0) | 1 | 0 (0, 0.1) | 4 | 0.2 (0, 0.5) |
| Other modern method | 0 | 0.0 (0.0, 0.0) | 0 | 0.0 (0.0, 0.0) | 0 | 0.0 (0.0, 0.0) |
| Collier | 0 | 0.0 (0.0, 0.0) | 0 | 0.0 (0.0, 0.0) | 0 | 0.0 (0.0, 0.0) |
| Unmet need for family planning |  |  |  |  |  |  |
| No | 775 | 66.2 (63, 69.5) | 1492 | 67.8 (65, 70.6) | 1964 | 69.1 (67.1, 71.1) |
| Yes | 364 | 33.8 (30.5, 37) | 638 | 32.2 (29.4, 35) | 862 | 30.9 (28.9, 32.9) |
| Modern use vs traditional or non use |  |  |  |  |  |  |
| Non user or traditional user | 953 | 87.2 (84.9, 89.4) | 1655 | 79 (75.8, 82.3) | 2125 | 76.4 (74.2, 78.5) |
| Modern contraceptive user | 186 | 12.8 (10.6, 15.1) | 475 | 21 (17.7, 24.2) | 701 | 23.6 (21.5, 25.8) |
| Long versus short acting contraceptive |  |  |  |  |  |  |
| Non user/traditional | 953 | 87.2 (84.9, 89.4) | 1655 | 79 (75.8, 82.3) | 2125 | 76.4 (74.2, 78.5) |
| LAPM | 5 | 0.3 (0, 0.5) | 27 | 0.9 (0.5, 1.4) | 94 | 2.9 (2.2, 3.6) |
| Short term methods | 181 | 12.6 (10.3, 14.8) | 448 | 20.1 (16.9, 23.2) | 607 | 20.8 (18.7, 22.9) |
