# Supplementary material for: Indicators of young women’s modern contraceptive use in Burkina Faso and Mali from Demographic and Health Survey data
Source: Contracept Reprod Med. 2017 Nov 3;2:26. doi: 10.1186/s40834-017-0053-6 (PMC5683538; doi:10.1186/s40834-017-0053-6)
Supplement: Supplementary file 2 — Mali: Distribution of variables of interest for sexually active women ages 15–24: Trends in the DHS Women’s Survey. This document includes a table of frequencies and weighted percentages for all variables of interest in Mali for the three most recent DHS surveys. (DOCX 34 kb) [file 40834_2017_53_MOESM2_ESM.docx]

*Mali: Distribution of variables of interest for sexually active women ages 15-24: Trends in the DHS Women’s Survey*

| **Variable Description** | **2001** | | **2006** | | **2012** | |
| --- | --- | --- | --- | --- | --- | --- |
|  | **Unweighted N = 2228** | | **Unweighted N = 2313** | | **Unweighted N = 1485** | |
|  | **Unweighted Frequency** | **Weighted Percent (95% CI)** | **Unweighted Frequency** | **Weighted Percent (95% CI)** | **Unweighted Frequency** | **Weighted Percent (95% CI)** |
| Wealth Index |  |  |  |  |  |  |
| Poorest | *N/A* | | 316 | 15.5 (12.5, 18.5) | 211 | 15.7 (12.3, 19.2) |
| Poorer |  |  | 341 | 14.6 (12.5, 16.8) | 225 | 14.9 (12.4, 17.4) |
| Middle |  |  | 421 | 17.9 (15.4, 20.4) | 211 | 14.9 (12.4, 17.5) |
| Richer |  |  | 492 | 18.8 (16.2, 21.3) | 340 | 25.4 (21.4, 29.3) |
| Richest |  |  | 743 | 33.2 (28.7, 37.6) | 498 | 29 (25.1, 33) |
| Age (mean and 95% CI) | 19.9 (19.7, 20) | | 19.9 (19.8, 20.1) | | 20 (19.9, 20.2) | |
| Educational attainment |  |  |  |  |  |  |
| No education | 1685 | 75.2 (72.2, 78.3) | 1537 | 67.9 (64.1, 71.7) | 869 | 59.6 (55.4, 63.9) |
| Primary | 258 | 12.2 (10.2, 14.1) | 306 | 13.1 (11, 15.1) | 163 | 11 (9.1, 12.9) |
| Secondary or higher | 285 | 12.6 (10, 15.2) | 470 | 19 (16.1, 22) | 453 | 29.4 (25.5, 33.3) |
| Urban/rural |  |  |  |  |  |  |
| Urban | 792 | 37.6 (31.8, 43.4) | 1052 | 42.4 (36.9, 47.9) | 572 | 30.7 (26.7, 34.6) |
| Rural | 1436 | 62.4 (56.6, 68.2) | 1261 | 57.6 (52.1, 63.1) | 913 | 69.3 (65.4, 73.3) |
| Currently working |  |  |  |  |  |  |
| No | 997 | 44 (39.7, 48.2) | 1117 | 45.2 (41.2, 49.2) | 949 | 64 (60.5, 67.4) |
| Yes | 1224 | 56 (51.8, 60.3) | 1188 | 54.8 (50.8, 58.8) | 536 | 36 (32.6, 39.5) |
| Religion |  |  |  |  |  |  |
| Muslim | 2083 | 95 (93.7, 96.3) | 2094 | 91 (89, 93) | 1381 | 92.9 (91, 94.9) |
| Christian | 52 | 2.6 (1.6, 3.6) | 77 | 3 (2, 4.1) | 63 | 4.2 (2.5, 5.8) |
| Animist/Traditional | 70 | 2.4 (1.5, 3.2) | 27 | 1.2 (0.6, 1.8) | 4 | 0.3 (0, 0.7) |
| No religion |  |  | 102 | 4.8 (3.4, 6.1) | 35 | 2.6 (1.5, 3.7) |
| Marital status |  |  |  |  |  |  |
| Never in union | 478 | 21.8 (18.2, 25.5) | 148 | 6.6 (4.5, 8.6) | 392 | 25.9 (21.9, 29.9) |
| Married | 1538 | 68.1 (64.2, 72) | 1474 | 64.2 (61.1, 67.3) | 957 | 65 (61, 69.1) |
| Living with partner | 104 | 5.2 (3.6, 6.7) | 543 | 23 (20, 26) | 94 | 6.2 (3.5, 8.9) |
| Widowed, Divorced or Separated | 108 | 4.9 (3.4, 6.4) | 148 | 6.2 (5, 7.4) | 42 | 2.8 (1.8, 3.9) |
| Health decision making |  |  |  |  |  |  |
| Husband alone or someone else | 1937 | 87.8 (85.9, 89.6) | 1999 | 86.6 (84.6, 88.7) | 908 | 87 (84.1, 89.9) |
| Respondent alone or with husband | 291 | 12.2 (10.4, 14.1) | 313 | 13.4 (11.3, 15.4) | 143 | 13 (10.1, 15.9) |
| Wife beating justified for refusal to have sex |  |  |  |  |  |  |
| No | 608 | 29 (25.8, 32.2) | 1094 | 44.5 (40.5, 48.5) | 624 | 41.7 (38.1, 45.3) |
| Yes | 1544 | 71 (67.8, 74.2) | 1142 | 55.5 (51.5, 59.5) | 834 | 58.3 (54.7, 61.9) |
| Home ownership |  |  |  |  |  |  |
| Does not own | *N/A* | | *N/A* | | 937 | 61.5 (56.8, 66.3) |
| Owns alone or jointly |  |  |  |  | 548 | 38.5 (33.7, 43.2) |
| Land ownership |  |  |  |  |  |  |
| Does not own | *N/A* | | *N/A* | | 1078 | 71.7 (67.4, 75.9) |
| Owns alone or jointly |  |  |  |  | 407 | 28.3 (24.1, 32.6) |
| Knowledge of contraceptive method |  |  |  |  |  |  |
| Knows no method | 445 | 17.3 (14.9, 19.7) | 477 | 18 (15.4, 20.6) | 151 | 10.4 (7.6, 13.1) |
| Knows only folkloric/traditional method | 23 | 1.3 (0.7, 2) | 17 | 0.8 (0.4, 1.2) | 2 | 0.1 (0, 0.3) |
| Knows modern method | 1760 | 81.4 (78.8, 84) | 1819 | 81.2 (78.5, 83.9) | 1332 | 89.5 (86.7, 92.3) |
| Heard FP on radio |  |  |  |  |  |  |
| No | 1103 | 46.8 (43.3, 50.3) | 1066 | 44.8 (41, 48.6) | 601 | 39.8 (35.5, 44) |
| Yes | 1125 | 53.2 (49.7, 56.7) | 1247 | 55.2 (51.4, 59) | 884 | 60.2 (56, 64.5) |
| Heard FP on TV |  |  |  |  |  |  |
| No | 1470 | 62.7 (59, 66.3) | 1317 | 56.8 (52.7, 60.8) | 703 | 48.5 (43.8, 53.2) |
| Yes | 757 | 37.3 (33.7, 41) | 996 | 43.2 (39.2, 47.3) | 782 | 51.5 (46.8, 56.2) |
| Heard FP in newspaper/magazine |  |  |  |  |  |  |
| No | 1906 | 85.4 (82.7, 88.1) | 2096 | 91.1 (89.4, 92.9) | 1404 | 94.6 (92.9, 96.2) |
| Yes | 320 | 14.6 (11.9, 17.3) | 211 | 8.9 (7.1, 10.6) | 81 | 5.4 (3.8, 7.1) |
| Visited by FP worker in last 12m |  |  |  |  |  |  |
| No | 2028 | 91.5 (89.9, 93.2) | 2090 | 90.8 (89.1, 92.6) | 1317 | 88.5 (86.2, 90.7) |
| Yes | 194 | 8.5 (6.8, 10.1) | 197 | 9.2 (7.4, 10.9) | 168 | 11.5 (9.3, 13.8) |
| Visited health facility in last 12m |  |  |  |  |  |  |
| No | 1472 | 65.6 (62.8, 68.3) | 1675 | 72.1 (69.2, 75.1) | 1057 | 71.9 (68.8, 74.9) |
| Yes | 753 | 34.4 (31.7, 37.2) | 616 | 27.9 (24.9, 30.8) | 428 | 28.1 (25.1, 31.2) |
| Barrier to getting medical help: permission to go |  |  |  |  |  |  |
| Big problem | 1815 | 82.6 (80.3, 84.9) | 430 | 18.9 (16.1, 21.7) | 410 | 27.1 (23.2, 31) |
| Small problem | 408 | 17.4 (15.1, 19.7) | 1881 | 81.1 (78.3, 83.9) | 1075 | 72.9 (69, 76.8) |
| Barrier to getting medical help: money |  |  |  |  |  |  |
| Big problem | 1171 | 53.7 (50.5, 56.8) | 1118 | 46.2 (42.3, 50.1) | 708 | 47.3 (43.4, 51.2) |
| Small problem | 1052 | 46.3 (43.2, 49.5) | 1191 | 53.8 (49.9, 57.7) | 777 | 52.7 (48.8, 56.6) |
| Barrier to getting medical help: distance |  |  |  |  |  |  |
| Big problem | 1401 | 63.2 (59.8, 66.6) | 829 | 33.9 (30.2, 37.6) | 471 | 32 (27.6, 36.3) |
| Small problem | 823 | 36.8 (33.4, 40.2) | 1481 | 66.1 (62.4, 69.8) | 1014 | 68 (63.7, 72.4) |
| Barrier to getting medical help: not wanting to go alone |  |  |  |  |  |  |
| Big problem | 1559 | 71.3 (68.4, 74.2) | 547 | 24.7 (21.9, 27.6) | 314 | 20.6 (17.5, 23.7) |
| Small problem | 666 | 28.7 (25.8, 31.6) | 1762 | 75.3 (72.4, 78.1) | 1171 | 79.4 (76.3, 82.5) |
| Number of other wives |  |  |  |  |  |  |
| No other wives | 1194 | 75.1 (72.5, 77.8) | 1513 | 77.3 (74.4, 80.1) | 830 | 79.7 (76.8, 82.5) |
| 1 other wife | 353 | 20.1 (17.5, 22.7) | 378 | 20.5 (17.9, 23.1) | 187 | 17.7 (15.2, 20.3) |
| 2-3 other wives | 86 | 4.6 (3.4, 5.9) | 47 | 2.3 (1.4, 3.1) | 23 | 2.6 (1.3, 3.9) |
| 4+ other wives | 2 | 0.1 (0, 0.3) | 0 | 0.0 (0.0, 0.0) | 0 | 0.0 (0.0, 0.0) |
| Age at first marriage/cohabitation (mean and 95% CI) | 15.5 (15.4, 15.7) | | 15.5 (15.4, 15.6) | | 15.6 (15.4, 15.7) | |
| Age at first sex (mean and 95% CI) | 15.2 (15.1, 15.3) | | 15.4 (15.3, 15.5) | | 15.5 (15.4, 15.6) | |
| Respondent has children |  |  |  |  |  |  |
| No | 548 | 25.4 (22, 28.8) | 538 | 23.4 (20.8, 25.9) | 365 | 23.9 (20.5, 27.4) |
| Yes | 1680 | 74.6 (71.2, 78) | 1775 | 76.6 (74.1, 79.2) | 1120 | 76.1 (72.6, 79.5) |
| Age at first birth (mean and 95% CI) | 17 (16.8, 17.1) | | 17 (16.8, 17.1) | | 16.6 (16.5, 16.8) | |
| Number of children (mean and 95% CI) | 1.7 (1.7, 1.8) | | 1.7 (1.6, 1.7) | | 1.8 (1.7, 1.8) | |
| Ideal number of children (mean and 95% CI) | 5.4 (5.3, 5.5) | | 5.4 (5.3, 5.6) | | 5.4 (5.3, 5.6) | |
| Respondent's desire for more children |  |  |  |  |  |  |
| Wants after 2+ years | 1313 | 60 (56.3, 63.7) | 1278 | 55 (51.9, 58.1) | 758 | 52.7 (49, 56.3) |
| Wants, unsure timing | 714 | 32.5 (29.2, 35.7) | 884 | 38.7 (35.6, 41.8) | 606 | 39.3 (35.5, 43) |
| Wants no more or can't have more | 201 | 7.5 (5.8, 9.3) | 151 | 6.3 (4.8, 7.8) | 121 | 8.1 (6.2, 9.9) |
| Husband's desire for more children |  |  |  |  |  |  |
| Both wants same | 250 | 16.3 (13.7, 18.9) | 436 | 22.8 (20, 25.6) | 184 | 17.6 (14.3, 21) |
| Husband wants more | 430 | 26.3 (22.9, 29.6) | 451 | 23.5 (20.4, 26.6) | 355 | 35.3 (30.7, 39.8) |
| Husband wants fewer | 49 | 3.5 (2.3, 4.7) | 43 | 2.8 (1.7, 3.9) | 34 | 3.1 (1.9, 4.3) |
| Don't know | 906 | 54 (49.4, 58.5) | 1071 | 50.9 (47.7, 54.1) | 478 | 44 (39.3, 48.7) |
| Ever terminated pregnancy |  |  |  |  |  |  |
| No | 2063 | 93.4 (92.1, 94.6) | 2172 | 93.2 (92, 94.4) | 1428 | 96.3 (95.2, 97.5) |
| Yes | 161 | 6.6 (5.4, 7.9) | 140 | 6.8 (5.6, 8) | 57 | 3.7 (2.5, 4.8) |
| Sex partners, last 12m |  |  |  |  |  |  |
| 0-1 partner | 2160 | 97.2 (96.3, 98.1) | 2255 | 97.2 (95.5, 98.9) | 1275 | 98.3 (97.6, 99) |
| 2+ Partners | 63 | 2.8 (1.9, 3.7) | 50 | 2.8 (1.1, 4.5) | 26 | 1.7 (1, 2.4) |
| Sex partners, lifetime |  |  |  |  |  |  |
| 1 partner | *N/A* | | 1764 | 76.2 (73.5, 78.8) | 1151 | 80.6 (77.9, 83.3) |
| 2 partners |  |  | 420 | 18.9 (16.6, 21.1) | 204 | 12.9 (10.6, 15.1) |
| 3+ partners |  |  | 119 | 5 (3.9, 6.1) | 95 | 6.5 (4.8, 8.2) |
| Recent sexual activity |  |  |  |  |  |  |
| Active in the last 4 weeks | 1197 | 55.6 (52.7, 58.4) | 1291 | 55.9 (53.2, 58.7) | 794 | 61.6 (57.9, 65.3) |
| Not active in last 4 weeks | 1026 | 44.4 (41.6, 47.3) | 1010 | 44.1 (41.3, 46.8) | 507 | 38.4 (34.7, 42.1) |
| Contraceptive use and intention |  |  |  |  |  |  |
| Using modern method | 209 | 9.4 (7.8, 11.1) | 265 | 10.2 (8.6, 11.8) | 246 | 15.3 (12.5, 18) |
| Using traditional method | 75 | 3.1 (2, 4.1) | 60 | 2.7 (1.8, 3.7) | 12 | 0.6 (0.2, 0.9) |
| Non user intends to use | 998 | 45.1 (41.4, 48.7) | 898 | 43.2 (39.7, 46.7) | 538 | 36.9 (32.5, 41.2) |
| Does not intend to use | 946 | 42.4 (39, 45.9) | 1090 | 43.9 (40.3, 47.4) | 689 | 47.3 (42.5, 52.1) |
| Last source of modern contraceptive |  |  |  |  |  |  |
| Government clinic/pharmacy | 92 | 44.5 (35.9, 53.1) | 101 | 37.4 (30.8, 44.1) | 173 | 70.5 (63, 78.1) |
| Private clinic/delivery | 11 | 6.9 (1.6, 12.2) | 24 | 12 (6.3, 17.7) | 19 | 7.1 (3.6, 10.5) |
| Pharmacy | 72 | 33.2 (25.1, 41.4) | 91 | 36 (29.4, 42.7) | 41 | 17.1 (10.8, 23.4) |
| Shop, church, friend | 25 | 13.9 (7.5, 20.3) | 34 | 9.8 (5.7, 13.9) | 12 | 5.3 (1, 9.7) |
| Other | 4 | 1.5 (0, 3.1) | 12 | 4.7 (1.7, 7.8) | 0 | 0.0 (0.0, 0.0) |
| Reasons for discontinuation of last method |  |  |  |  |  |  |
| Became pregnant | *N/A* | | *N/A* | | 10 | 11.3 (4, 18.6) |
| Wanted to become pregnant |  |  |  |  | 25 | 24.1 (14.5, 33.6) |
| Husband disapproved |  |  |  |  | 5 | 3.7 (0.3, 7.1) |
| Side effects |  |  |  |  | 28 | 21.4 (13.6, 29.3) |
| Access, availability |  |  |  |  | 1 | 1.5 (0, 4.3) |
| Wanted more effective method |  |  |  |  | 7 | 5.9 (1.3, 10.4) |
| Inconvenient to use |  |  |  |  | 3 | 3.6 (0, 7.8) |
| Infrequent sex, husband away |  |  |  |  | 11 | 10.7 (4.3, 17.2) |
| Cost |  |  |  |  | 1 | 1.3 (0, 3.9) |
| Fatalistic |  |  |  |  | 2 | 1.5 (0, 3.5) |
| Difficult pregnancy, menopause |  |  |  |  | 2 | 2.3 (0, 5.8) |
| Marital dissolution |  |  |  |  | 0 | 0.0 (0.0, 0.0) |
| Other |  |  |  |  | 11 | 10.5 (3.7, 17.3) |
| Don't know |  |  |  |  | 4 | 2.2 (0, 4.5) |
| Condom use at last sex |  |  |  |  |  |  |
| No | 1834 | 95.1 (93.9, 96.4) | 1916 | 94.2 (92.9, 95.5) | 1108 | 92.7 (90.6, 94.8) |
| Yes | 103 | 4.9 (3.6, 6.1) | 131 | 5.8 (4.5, 7.1) | 87 | 7.3 (5.2, 9.4) |
| Current contraceptive method |  |  |  |  |  |  |
| Not using | 1944 | 87.5 (85.4, 89.6) | 1988 | 87.1 (85, 89.2) | 1227 | 84.2 (81.4, 86.9) |
| Pill | 90 | 3.9 (2.8, 5) | 116 | 4.2 (3.3, 5.2) | 63 | 4.1 (2.8, 5.3) |
| IUD | 4 | 0.2 (0, 0.5) | 1 | 0 (0, 0.1) | 9 | 0.4 (0, 0.7) |
| Injections | 71 | 3 (2.1, 3.9) | 96 | 4 (3, 5) | 101 | 6.4 (5, 7.8) |
| Diaphragm | 0 | 0.0 (0.0, 0.0) | 0 | 0.0 (0.0, 0.0) | 0 | 0.0 (0.0, 0.0) |
| Condom | 42 | 2.2 (1.4, 3.1) | 49 | 1.9 (1.2, 2.5) | 7 | 0.6 (0, 1.4) |
| Male sterilization | 0 | 0.0 (0.0, 0.0) | 0 | 0.0 (0.0, 0.0) | 0 | 0.0 (0.0, 0.0) |
| Periodic abstinence | 56 | 2.4 (1.4, 3.3) | 42 | 1.8 (1.1, 2.6) | 3 | 0.1 (0, 0.3) |
| Withdrawl | 0 | 0.0 (0.0, 0.0) | 2 | 0.2 (0, 0.4) | 0 | 0.0 (0.0, 0.0) |
| Other traditional | 19 | 0.7 (0.3, 1) | 16 | 0.7 (0.3, 1.1) | 8 | 0.4 (0.1, 0.6) |
| Implants/norplant | 1 | 0 (0, 0.1) | 2 | 0 (0, 0.1) | 65 | 3.7 (2.4, 5.1) |
| Abstinence | 0 | 0.0 (0.0, 0.0) | 0 | 0.0 (0.0, 0.0) | 0 | 0.0 (0.0, 0.0) |
| Female condom | 0 | 0.0 (0.0, 0.0) | 0 | 0.0 (0.0, 0.0) | 0 | 0.0 (0.0, 0.0) |
| Foam or jelly | 1 | 0 (0, 0.1) | 1 | 0.1 (0, 0.2) | 0 | 0.0 (0.0, 0.0) |
| Other modern method | 0 | 0.0 (0.0, 0.0) | 0 | 0.0 (0.0, 0.0) | 1 | 0.1 (0, 0.2) |
| Collier | 0 | 0.0 (0.0, 0.0) | 0 | 0.0 (0.0, 0.0) | 1 | 0.1 (0, 0.2) |
| Unmet need for family planning |  |  |  |  |  |  |
| No | 1285 | 57.8 (54.7, 60.8) | 1264 | 54.1 (50.9, 57.4) | 538 | 50.2 (45.2, 55.2) |
| Yes | 943 | 42.2 (39.2, 45.3) | 1049 | 45.9 (42.6, 49.1) | 473 | 49.8 (44.8, 54.8) |
| Modern use vs traditional or non use |  |  |  |  |  |  |
| Non user or traditional user | 2019 | 90.6 (88.9, 92.2) | 2048 | 89.8 (88.2, 91.4) | 1239 | 84.7 (82, 87.5) |
| Modern contraceptive user | 209 | 9.4 (7.8, 11.1) | 265 | 10.2 (8.6, 11.8) | 246 | 15.3 (12.5, 18) |
| Long versus short acting contraceptive |  |  |  |  |  |  |
| Non user/traditional | 2019 | 90.6 (88.9, 92.2) | 2048 | 89.8 (88.2, 91.4) | 1239 | 84.7 (82, 87.5) |
| LAPM | 5 | 0.3 (0, 0.5) | 3 | 0 (0, 0.1) | 74 | 4.1 (2.8, 5.5) |
| Short term methods | 204 | 9.2 (7.6, 10.8) | 262 | 10.1 (8.5, 11.8) | 172 | 11.2 (9.1, 13.2) |
