# Supplementary material for: Indicators of young women’s modern contraceptive use in Burkina Faso and Mali from Demographic and Health Survey data
Source: Contracept Reprod Med. 2017 Nov 3;2:26. doi: 10.1186/s40834-017-0053-6 (PMC5683538; doi:10.1186/s40834-017-0053-6)
Supplement: Supplementary file 3 — Binary outcome analysis. This document includes a description of preliminary significance testing using a binary outcome to limit variables for model inclusion in the multivariate analysis. (DOCX 18 kb) [file 40834_2017_53_MOESM3_ESM.docx]

## Binary Outcome Analysis

In Burkina Faso, age, attaining secondary or higher education, owning a home, having multiple lifetime sex partners, and being sexually active in the last 4 weeks, increased the odds of using modern contraceptives for young women regardless of if they were married (p≤0.05) [Table 5]. Completing primary education and seeing family planning messaging on TV also increased the odds of modern contraceptive use for all women in the sample (p≤0.05), however, this association did not exist when limited to married women. For all women, living with a partner increased the odds of using a modern contraceptive (p≤0.05) compared to married women.

Having children, being from a rural area, and larger ideal family size decreased the odds of using a modern contraceptive (p≤0.05). For married women whose partner’s wanted more children had 40% lower odds of using modern contraceptives (p≤0.05).

## Burkina Faso 2010: Variables showing evidence of association with modern contraceptive use

|  | | |
| --- | --- | --- |
|  | **All women, ages 15-24,**  **N=2,826**  **OR (95% CI)** | **Married women, ages 15-24,**  **N=2,117**  **OR (95% CI)** |
| Wealth Index (standardized) | 1.1 (0.9, 1.2) | 1.4 (1, 1.8)* |
| Age | 1.1 (1, 1.1)* | 1.1 (1, 1.2)* |
| Educational attainment |  |  |
| No education | REF | REF |
| Primary | 1.4 (1.1, 1.9)* | 1.1 (0.7, 1.6) |
| Secondary or higher | 2.7 (1.9, 3.9)* | 2.2 (1.2, 4)* |
| Urban/rural |  |  |
| Urban | REF | REF |
| Rural | 0.6 (0.4, 0.8)* | 0.6 (0.4, 1)* |
| Home ownership |  |  |
| Does not own | REF | REF |
| Owns alone or jointly | 1.8 (1.3, 2.6)* | 1.6 (1.1, 2.4)* |
| Marital status |  | *N/A* |
| Married | REF |  |
| Never in union | 1.7 (1, 2.9) |  |
| Living with partner | 1.8 (1.2, 2.9)* |  |
| Widowed/Divorced/Separated | 1.1 (0.4, 3.2) |  |
| Saw FP messages on TV |  |  |
| No | REF | REF |
| Yes | 1.4 (1, 2)* | 1.3 (0.8, 2.1) |
| Respondent has children |  |  |
| No | REF | REF |
| Yes | 0.6 (0.4, 0.9)* | 0.4 (0.2, 0.8)* |
| Ideal number of children | 0.9 (0.8, 1)* | 0.8 (0.8, 1)* |
| Husband's desire for more children | *N/A – marriage*  *specific variable* |  |
| Both wants same |  | REF |
| Husband wants more |  | 0.6 (0.4, 0.8)* |
| Husband wants fewer |  | 1.5 (0.6, 3.6) |
| Don't know |  | 0.6 (0.4, 1) |
| Sex partners, lifetime |  |  |
| 1 partner | REF | REF |
| 2 partners | 1.5 (1.1, 2.1)* | 1.7 (1.2, 2.6)* |
| 3+ partners | 2.1 (1.2, 3.4)* | 2 (1.0, 3.9)* |
| Recent sexual activity |  |  |
| Active in the last 4 weeks | 6.2 (4.7, 8.3)* | 9.5 (6.4, 14.1)* |
| Not active in last 4 weeks | REF | REF |

†Adjusted for the following variables: wealth index (standardized), currently working, land ownership, religion, wife beating justified for refusal to have sex, saw FP messages in newspaper/magazine, barrier to getting medical help: money, barrier to getting medical help: distance, respondent’s desire for more children, number of other wives, sex partners in last 12m
*p<0.05

In Mali, age, secondary or higher educational attainment, wanting another child in 2+ years and visiting a health facility in the last 12 months increased the odds of using modern contraceptives for all women and married women (p≤0.05). For all women, having been sexually active over the last four weeks and having two lifetime sex partners increased their odds of using modern contraceptives (p≤0.05), however, this relationship did not exist when the analysis was restricted to married women.

Increasing the ideal number of children a woman wants to have lowered the odds of her using a modern contraceptive, regardless of marital status (p≤0.05). Women who own a home also had lower odds of using modern contraceptives (p≤0.05). The odds of using modern contraceptives for married women whose husband wanted more children than they did was 70% less than those who wanted the same number of children as their husbands (p≤0.05) [Table 6].

## Mali 2012: Variables showing evidence of association with modern contraceptive use

|  | **All women, ages 15-24, N = 1,485**  **OR (95% CI)** | **Married women, ages 15-24, N = 1,093**  **OR (95% CI)** |
| --- | --- | --- |
| Age | 1.1 (1, 1.2)* | 1.2 (1, 1.3)* |
| Educational attainment |  |  |
| No education | REF | REF |
| Primary | 0.9 (0.5, 1.7) | 1 (0.5, 2.2) |
| Secondary or higher | 2.4 (1.4, 4.2)* | 2.9 (1.6, 5.4)* |
| Home ownership |  |  |
| Does not own | REF | REF |
| Owns alone or jointly | 0.7 (0.4, 1)* | 0.6 (0.3, 0.9)* |
| Visited health facility in last 12m |  |  |
| No | REF | REF |
| Yes | 1.8 (1.2, 2.7)* | 1.7 (1, 2.8)* |
| Respondent's desire for more children |  |  |
| Wants after 2+ years | 2.5 (1.1, 5.7)* | 4.8 (1.4, 16.8)* |
| Wants, unsure timing | 2.3 (0.9, 5.7) | 1.5 (0.3, 6.7) |
| Wants no more or can't have more | REF | REF |
| Ideal number of children | 0.8 (0.7, 0.9)* | 0.8 (0.7, 0.9)* |
| Husband's desire for more children | *N/A* |  |
| Both wants same |  | REF |
| Husband wants more |  | 0.3 (0.1, 0.6)* |
| Husband wants fewer |  | 0.5 (0.1, 2) |
| Don't know |  | 0.4 (0.2, 0.8)* |
| Sex partners, lifetime |  |  |
| 1 partner | REF | REF |
| 2 partners | 1.8 (1.1, 2.9)* | 1.7 (0.9, 3.2) |
| 3+ partners | 1.8 (0.9, 3.5) | 1 (0.3, 3) |
| Recent sexual activity |  |  |
| Active in the last 4 weeks | 1.9 (1.3, 2.8)* | 1.5 (0.9, 2.5) |
| Not active in last 4 weeks | REF | REF |

†Adjusted for the following variables: currently working, religion, saw FP messages on TV, barrier to getting medical help: not wanting to go alone, respondent has children, number of other wives
*p<0.05
